# Road Traffic Injuries and Related Safety Measures: A Multicentre Analysis at Military Hospitals in Tabuk, Saudi Arabia

**DOI:** 10.1155/2021/6617381

**Published:** 2021-02-10

**Authors:** Umar Yagoub, Nasrin S. Saiyed, Bahaa-eldin E. A. Rahim, Nizar Musawa, Attiya Mohammed Al Zahrani

**Affiliations:** ^1^Research Department, King Salman Armed Forces Hospital, Tabuk 71411, Saudi Arabia; ^2^Department of Academic Affairs, King Salman Armed Forces Hospital, Tabuk 71411, Saudi Arabia; ^3^Department of Surgery, King Salman Armed Forces Hospital, Tabuk 71411, Saudi Arabia

## Abstract

**Background:**

Road traffic injuries are one of the major public safety issues around the world, as they put a great burden on countries' economies. Saudi Arabia has a good road transportation system, but still, road-related fatalities are higher in this country. The main purpose of this study was to determine the association between the severity of injuries caused by road traffic collision and safety measures taken by the drivers admitted to the emergency departments at two military hospitals in Tabuk, a city in Saudi Arabia.

**Methods:**

A total of 342 male drivers who had injuries due to road traffic collision took part in this multicentre, cross-sectional study. The research sites were King Salman Armed Forces Hospital and King Khalid Armed Forces Hospital in Tabuk, Saudi Arabia. The data were collected using a common protocol and a self-reported questionnaire with the help of convenience sampling approach. Descriptive statistics and logistic regression analysis were done using IBM SPSS version 23.

**Results:**

Nearly 62.0% of the male drivers were aged less than 36 years. About 90% of the drivers were from low- and middle-income groups. Logistic regression analysis indicated that drivers who had road traffic collision and used mobile phones while driving were four times more likely to have severe injuries (OR = 3.89; 95% CI (2.53, 5.95)), those who drove the vehicle at an excessive speed limit were three times more likely to have severe injuries (OR = 2.71; 95% CI (1.01, 4.97)), and those who attempted to overtake another vehicle were two times more likely to have severe injuries (OR = 1.85; 95% CI (1.24, 3.77)).

**Conclusion:**

Based on the results of the present study, the most important safety measures which contributed the most to road traffic collision were use of mobile phones while driving the vehicle, driving at an excessive speed limit, irregularity in maintaining the vehicle, attempt to pass other vehicles, and not following the traffic rules. All the safety measures are protective, but if care is not taken, they will risk the life. There is an urgent need to spread traffic safety awareness in this region.

## 1. Introduction

Road traffic safety has a significant impact on public health. Rapid motorisation in developing countries, along with the poor quality of road traffic systems and lack of government capacity to manage outcomes, is contributing to a growing road traffic collision [1, 2]. In 2018, the World Health Organisation (WHO) published a worldwide road traffic safety report, which highlighted that the yearly fatalities caused by road crashes had risen to 1.35 million in a year. In recent times, road traffic injuries (RTIs) are the common cause of fatality among young adults aged 5–29 years. The prime victims of road traffic collision are pedestrians, motorists, and cycle riders [3]. The casualties related to road traffic collision are high in the Kingdom of Saudi Arabia, as four-wheel automobiles are the main means of transportation. Saudi Arabia ranked first among Arab countries regarding the road accident fatality rate [4].

The Saudi Arabian government is constantly making efforts to decrease the number of deaths due to road injuries. The Ministry of Interior, Saudi Arabia, has set a target to reduce road traffic deaths to six per 100,000 as a part of the government's Vision 2030 program. There is a need to study the safety measures taken by the drivers associated with road traffic injuries. The information obtained from this study could be adopted by the policymakers and key stakeholders. It can serve as the first step toward addressing these critical issues and elements and improving safety programs and enhanced road policies. The main aim of this study was to look for an association between the severity of injuries due to road traffic collision and safety measures taken by the adult male drivers who were hospitalized and survived after road traffic collision.

## 2. Materials and Methods

### 2.1. Study Population and Study Design

This study was carried out using a cross-sectional design. Two accident and emergency departments, at King Salman Armed Forces Hospital and King Khalid Military Hospital, were used as the study settings. This study was conducted using a common protocol from January 2018 to July 2018. The study population for this research was adult male drivers aged 18 years and above who were hospitalized following a road traffic collision. Before conducting the actual study, a pilot survey was conducted for one month to evaluate the study population, research sites, and reliability of the data.

### 2.2. Sample Size and Sampling Procedure

The adequate sample size for this survey was estimated using the following statistical formula:

(1) $$\begin{array}{c} n=\frac{Z^{2}*p\left(1-p\right)}{d^{2}}. \end{array}$$

The parameters used for this calculation were as follows: *α* = level of significance = 5%, *Z* = standard normal score at confidence level 100(1−*α*)% = 1.96, *p* = anticipated prevalence of incidence of road traffic injuries taken as 50% in order to achieve the maximum sample size, and*d* = absolute precision 5%. Considering these parameters, the approximate sample size turned out to be 384. Assuming a 10% nonresponse rate, the revised sample size was 423. A convenience sampling technique was used in the selection process. A total of 425 male drivers who had a road traffic collision were asked to answer questionnaires, out of which 342 willingly and voluntarily participated in the study with a response rate of 80.4%.

### 2.3. Study Tool Development and Its Validity

The questionnaire for this study was developed using an extensive literature review. The study questionnaire was developed in English and Arabic languages by bilingual experts. It was categorised into the following sections: the participants' sociodemographic details, vehicle ownership, everyday driving activity, traffic rules violations, history of accident exposure, and precautionary measures taken by the participants. The road traffic injuries of the crash victims were categorised as severe injuries and not severe injuries. The study tool was prepared in a fair length with a single statement for every question in order to achieve good response rate. The content validity of the study tool was assessed by three subject experts. The study tool's internal consistency was determined by using the reliability coefficient Cronbach's alpha (*α* = 0.87).

### 2.4. Survey Administration

The well-trained data collectors interviewed drivers before the hospital discharge. The interviews took place when the participants were in a stable condition to answer the survey questions. The type of injuries among victims and severity were reported by attending doctors at the accident and emergency departments. The interviews were in English and Arabic languages for both Arabic and non-Arabic speaking drivers. The mean time to fill out the survey form was approximately 15–20 minutes. The drivers and their accompanied individuals were informed about the study purpose, voluntary participation, and confidentiality of the research data. Informed consent was obtained from the participants.

### 2.5. Data Management and Statistical Analysis

A double data entry verification method was used to ensure high-quality data. The statistical data analysis was done using IBM SPSS version 23.0 for Windows. Descriptive statistics of the data were computed, which included frequency and percentages. A binary logistic regression model was used to predict the association between the severity of road traffic injuries (dependent variable) and safety measures taken by the drivers (set of independent variables). Inclusion of these safety measures in the model depended on the significance of the correlation with the severity of the injuries. The adjusted odds ratios (ORs) and 95% confidence interval (CI) were calculated. Statistical significance was indicated by a *P*-value <0.05.

## 3. Results

### 3.1. Sociodemographic Characteristics of the Study Participants

During the study period, 342 male drivers who had injuries due to road traffic collision participated. The sociodemographic characteristics of the study participants are shown in Table 1. Nearly 62% of the drivers were less than 36 years old. More than two thirds were living in an urban area (68.1%). Most were married (80.4%), had a high school or university education (70.8%), and were employed (82.7%). The majority of the drivers were from low- and middle-income group (88.9%).

### 3.2. Vehicle Ownership, Driving History, and Other Related Particulars

Table 2 shows the vehicle ownership, driving history, and other related particulars of the participants. Of the 342 participants, 90.9% owned a vehicle. Nearly seven out of ten (64.9%) had more than five years of driving experience. The majority of the participants (84.0%) drove their vehicles 10–30 kilometres per day. Approximately seven out of ten participants drove their vehicles at 61–80 kilometres per hour in the city (68.1%) and 101–120 kilometres per hour on highways (69.6%). About 42% of the participants had made a traffic violation in the past.

### 3.3. Road Traffic Injuries and Related Particulars

Table 3 illustrates the types of road traffic injuries and other related particulars of the participants. Of the total study sample, 61.1% reported that they had no previous experience of a road traffic collision. When the participants were asked about the frequency of the past road traffic collision, one of four (24.9%) participants had more than one incident of the same. Six of ten (62.0%) participants had a vehicle-to-vehicle collision. About 32% of the participants reported that use of a mobile phone was the main reason for their current road traffic collision. About 33.0% of the participants had head and/or neck injuries. Approximately 30% of the participants had severe injuries.

### 3.4. Logistic Regression Model Predicting the Severity of Injuries Using Safety Measures Taken by Drivers

Table 4 represents the output of logistic regression model predicting the severity of injuries using safety measures taken by the drivers who had road traffic injuries. The analysis indicated that the fitted model was good with *χ*^2^ = 689.63 and *P*-value = 0.782 (deviance criterion used), with the coefficient of determination Nagelkerke *R*^2^ = 0.642. Moreover, the overall significance test of full model against the intercept-only model showed a significant result, *χ*^2^ = 763.43, *P*-value <0.0001, suggesting that the safety measures mentioned hereinafter as a whole satisfactorily predict the severity of the injuries among the drivers who experienced road traffic collision.

The drivers who did not maintain their vehicles regularly, did not fasten their seatbelts, and did not follow lane discipline were 1.5 times more likely to have severe injuries. The drivers who did not obey traffic lights were more prone to have severe injuries. The drivers who did not drive within the specified speed limit were 2.7 times more likely to have severe injuries. The drivers who used a mobile phone while driving were 3.8 times more likely to have severe injuries.

## 4. Discussion

Road traffic collisions can cause severe injuries and fatalities, which leave a negative impact on the mental and physical health, social life, and financial conditional of the victims and their families [5–7]. Road traffic injuries are a big concern for government authorities and the general public in the Kingdom of Saudi Arabia [8, 9]. Many studies have examined the relationship between road traffic collisions and drivers' personal characteristics, such as age, sex, country of origin, and driving history [10–12]. However, very few had studied the safety measures taken by drivers who had road traffic injuries. The present study was conducted to determine the association between the severity of road traffic injuries and safety measures taken by the drivers who experienced road traffic collisions.

The age of the driver is a significant factor associated with road traffic injuries. International studies have shown that young drivers aged less than 40 years were more prone to have road traffic injuries [13–15]. A similar observation which was found in the present study is that most of the drivers who had road traffic injuries were less than 36 years old. This observation was also reported by other studies conducted in developed and developing countries [16–20]. Cities are more populated compared with the rural areas in the Saudi Arabia. The results of the present study revealed that the majority of drivers who had road traffic injuries lived in urban areas. This result is consistent with that of other studies [21–23]. In the current study, it was observed that road traffic collisions were high among the educated drivers. This observation is in line with other studies [12, 24]. On the contrary, studies conducted in other countries showed that drivers who did not have a much higher education were more likely to be involved in road traffic injuries [25, 26].

Driving experience is another contributing factor to road traffic collisions [23]. This study revealed that about seven out of ten crash victims had a driving experience of over five years. Similar findings have been mentioned in the study conducted in Tabuk city [12]. Studies have also found a relationship between the prevalence of road crashes and vehicles' speed. In the current study, it was noted that approximately one of five crash victims had driven their vehicles at speeds of more than 80 kilometres per hour in the city. These results are consistent with other studies conducted in the Kingdom of Saudi Arabia [27]. Obeying road traffic regulations also plays a major role in preventing road traffic fatalities. In this study, two of five victims of crashes had been in road injuries or had had traffic violations in the past [27]. Also, in this study, six of ten victims had had a vehicle-to-vehicle collision. About 33% of crash victims suffered head and/or neck injuries. This result was in accordance with those of other regional and international studies [28, 29]. When victims were asked about the reason for the current crash, they reported that excessive speed or use of a mobile phone was the major reason. Similar findings have been reported by many other national and international studies [30, 31].

Regular maintenance of the vehicle is important in preventing road traffic injuries. The victims who did not maintain their vehicle regularly, did not fasten their seatbelts, and did not follow lane discipline were 1.5 times more likely to have had severe injuries. These results are in agreement with other studies [32, 33]. Most road traffic injuries occurred due to texting while driving. Studies have reported that distracted drivers were more prone to having a crash. In this study, the subjects who used mobile phones had a 3.8 times higher chance of having severe injuries. This result is supported by studies conducted in higher-income countries as well as middle-income countries [30, 31]. Drivers who do not follow the rules of the road are more likely to have crashes. The result of the present study revealed that crash victims who did not obey traffic lights were 1.6 times more likely to have severe injuries, those who drove at an excessive speed limit were 2.4 times more likely to have severe injuries, those who attempted to pass other vehicles were 1.8 times more likely to have severe injuries, and those who changed lanes suddenly were 1.2 times more likely to have severe injuries. These findings are in accordance with research carried out in various parts of the world [14, 19, 20, 34].

## 5. Strengths and Limitations of This Study

This research was the first of its kind in the Tabuk region to use an appropriate statistical approach to study the safety measures taken by the male drivers who had road injuries. There is some potential recall bias, as the analysis in the study was based on self-reported data. Despite official lifting of ban on women driving on 24 June 2018, female drivers were excluded from the study due to cultural/social restrictions on women driving in this conservative seminomadic part of Saudi Arabia. Only the male drivers were included in the present study.

## 6. Conclusion

The safety measures taken by the drivers were interpreted in light of the severity of injuries caused by road traffic collision. The important safety measures were the use of a cell phone while driving, driving at excessive speed limit, irregularity in maintaining the vehicle, attempt to pass other vehicles, and not following the traffic rules. This study calls for a need to spread traffic safety awareness among drivers in the city of Tabuk in the northwest region of Kingdom of Saudi Arabia.

## 7. Recommendations

This study stressed the need for continuous educational initiatives to develop and encourage a sound driving culture and sound attitudes among adolescents and young adults at the tertiary educational level, through lectures and workshops. In particular, the implementation of more road safety camera systems along highways would effectively curb the most common risky driving habits, such as speeding and cell phone use. Establishing various high-capacity modes of public transport, particularly in large cities, can be an effective intervention from a strategic perspective to reduce the rate of road injuries and subsequently long-term road traffic injuries. Finally, it is important to mention that as this study is the first of its kind, this will serve as the baseline study for further on-site observational research. A large cohort study research is needed to analyse other contributing factors to road crashes.

## Figures and Tables

**Table 1:** Sociodemographic characteristics of the study participants (*n* = 342).

Sociodemographics	Categories	Frequency (*n*)	Percentage (%)
Age	18–25 years	120	35.1
	26–35 years	92	26.9
	36–45 years	83	24.3
	≥46 years	47	13.7
	Total	**342**	**100**

Place of residence	Rural	109	31.9
	Urban	233	68.1
	Total	**342**	**100**

Marital status	Unmarried	53	15.5
	Married	275	80.4
	Widowed	2	0.6
	Divorced	7	2.0
	Separated	5	1.5
	Total	**342**	**100**

Education	No schooling	14	4.1
	Elementary school	33	9.6
	Secondary school	53	15.5
	Higher secondary school	136	39.8
	College/university	106	31.0
	Total	**342**	**100**

Employment	Student	32	9.4
	Unemployed	15	4.4
	Employed	283	82.7
	Retired	12	3.5
	Total	**342**	**100**

Monthly income	≤10,000 SAR	105	30.7
	10,001–15,000 SAR	199	58.2
	>15,000 SAR	38	11.1
	Total	**342**	**100**

SAR: Saudi Arabian Riyal.

**Table 2:** Vehicle ownership, driving history, and other related particulars (*n* = 342).

Vehicle ownership, driving history, and other related particulars	Categories	Frequency (*n*)	Percentage (%)
Vehicle ownership	Yes	311	90.9
	No	31	9.1
	Total	**342**	**100**

Driving experience	Less than 5 years	120	35.1
	5–10 years	130	38.0
	More than 10 years	92	26.9
	Total	**342**	**100**

Driving distance per day	Less than 10 km	27	7.9
	10–30 km	287	84.0
	More than 30 km	28	8.1
	Total	**342**	**100**

Vehicle speed in the city	≤40 kmph	7	2.0
	41–60 kmph	31	9.1
	61–80 kmph	233	68.1
	More than 80 kmph	71	20.8
	Total	**342**	**100**

Vehicle speed on highways	81–100 kmph	68	19.9
	101–120 kmph	238	69.6
	More than 120 kmph	36	10.5
	Total	**342**	**100**

Traffic violation received in the past	Yes	145	42.4
	No	197	57.6
	Total	**342**	**100**

km: kilometres; kmph: kilometres per hour.

**Table 3:** Road traffic injuries and related particulars (*n* = 342).

Road traffic injuries and related particulars	Categories	Frequency (*n*)	Percentage (%)
Involvement of road traffic collision in the past	Yes	133	38.9
	No	209	61.1
	Total	**342**	**100**

Number of road traffic collisions in the past	None	209	61.1
	1 time	48	14.0
	2 times	42	12.3
	≥3 times	43	12.6
	Total	**342**	**100**

Type of current road traffic collision	Vehicle–vehicle collision	212	62.0
	Vehicle–fixed object collision	27	7.9
	Vehicle–pedestrian collision	35	10.2
	Vehicle–animal collision	7	2.0
	Overturning of the vehicle	41	12.0
	Vehicle out of control	17	5.0
	Other types of collision	3	0.9
	Total	**342**	**100**

Reasons for current road traffic collision	Excessive speed limit	85	24.9
	Disobeying traffic signals	41	12.0
	Incorrect passing	34	9.9
	Sudden lane change	23	6.7
	Use of mobile phone	109	31.9
	Vehicle condition	17	5.0
	Effects of the weather	7	2.0
	Others	26	7.6
	Total	**342**	**100**

Anatomical region of road traffic injuries	Head and/or neck	113	33.0
	Thorax	21	6.1
	Abdomen	27	7.9
	Pelvic	31	9.1
	Upper limbs	34	9.9
	Lower limbs	44	12.9
	Spinal cord	14	4.1
	Others	58	17.0
	Total	**342**	**100**

Severe injuries	Yes	103	30.1
	No	239	69.9
	Total	**342**	**100**

**Table 4:** Binary logistic regression model predicting the severity of injuries using safety measures taken by road crash victims (*n* = 342).

Safety measures taken by drivers	*β*	OR adjusted	OR, 95% CI	*P*-value
Did not regularly maintain the vehicle	1.124	1.53	(1.23, 2.90)	0.01^*∗*^
Did not fasten safety seatbelts	1.328	1.52	(1.20, 2.68)	0.01^*∗*^
Did not followed lane discipline	1.627	1.53	(1.17, 2.98)	0.02^*∗*^
Disobeyed traffic lights	1.960	1.61	(1.04, 1.97)	0.04^*∗*^
Did not drive within the specified speed limit	1.463	2.71	(1.01, 4.97)	0.004^*∗*^
Used a mobile phone	1.502	3.89	(2.53, 5.95)	0.001^*∗*^
Ate/drank/smoked/played music	0.963	0.54	(0.34, 0.87)	0.072
Drove for a prolonged time	0.746	0.87	(0.70, 1.09)	0.367
Made a sudden lane change	1.362	1.28	(1.06, 1.54)	0.008^*∗*^
Attempted to pass other vehicles	1.443	1.85	(1.24, 2.77)	0.003^*∗*^
Crossed the traffic light at the last moment	1.364	0.92	(0.72, 1.16)	0.235
Drove in bad weather conditions	0.964	0.53	(0.32, 0.78)	0.074

Reference category: no. ^*∗*^A significant *P*-value. OR: odds ratio; CI: confidence interval.

## Data Availability

The datasets used and/or analysed during the current study are available from the corresponding author on reasonable request.
